# Feasibility, utility, and safety of fully incorporating transesophageal echocardiography into emergency medicine practice

**DOI:** 10.1111/acem.14399

**Published:** 2021-11-06

**Authors:** Robert F. Reardon, Elliott Chinn, Dave Plummer, Andrew Laudenbach, Andie Rowland Fisher, Will Smoot, Daniel Lee, Joseph Novik, Barrett Wagner, Chris Kaczmarczyk, Johanna Moore, Emily Thompson, Craig Tschautscher, Teresa Dunphy, Thomas Pahl, Michael A. Puskarich, James R. Miner

**Affiliations:** ^1^ Department of Emergency Medicine Hennepin County Medical Center Minneapolis Minnesota USA; ^2^ Glacial Ridge Health System Glenwood Minnesota USA; ^3^ Department of Emergency Medicine Hennepin County Medical Center & University of Minnesota Medical School Minneapolis Minnesota USA

## Abstract

**Introduction:**

Transthoracic echocardiography (TTE) is a standard procedure for emergency physicians (EPs). Transesophageal echocardiography (TEE) is known to have great utility in patients who are critically ill or in cardiac arrest and has been used by some EPs with specialized ultrasound (US) training, but it is generally considered outside the reach of the majority of EPs. We surmised that all of our EPs could learn to perform focused TEE (F‐TEE), so we trained and credentialed all of the physicians in our group.

**Methods:**

We trained 52 EPs to perform and interpret F‐TEEs using a 4‐h simulator‐based course. We kept a database of all F‐TEE examinations for quality assurance and continuous quality feedback. Data are reported using descriptive statistics.

**Results:**

Emergency physicians attempted 557 total F‐TEE examinations (median = 10, interquartile range = 5–15) during the 42‐month period following training. Clinically relevant images were obtained in 99% of patients. EPs without fellowship or other advanced US training performed the majority of F‐TEEs (417, 74.9%) and 94.3% (95% confidence interval [CI] = 91.4%–96.3%) had interpretable images recorded. When TTE and TEE were both performed (*n* = 410), image quality of TEE was superior in 378 (93.3%, 95% CI = 89.7%–95%). Indications for F‐TEE included periarrest states (55.7%), cardiac arrest (32.1%), and shock (12.2%). There was one case of endotracheal tube dislodgement during TEE placement, but this was immediately identified and replaced without complication.

**Conclusion:**

After initiating a mandatory group F‐TEE training and credentialing program, we report the largest series to date of EP‐performed resuscitative F‐TEE. The majority of F‐TEE examinations (75%) were performed by EPs without advanced US training beyond residency.

## INTRODUCTION

Point‐of‐care (POC) echocardiography was described by emergency physicians (EPs) 30 years ago and is now a well‐accepted component of emergency medicine (EM) practice and a requirement for EM residency training.^1^, ^2^, ^3^, ^4^, ^5^, ^6^, ^7^, ^8^ Several factors, including suboptimal positioning, difficult body habitus, and inaccessibility of the chest (during cardiopulmonary resuscitation [CPR]) often make it difficult to obtain interpretable images when using transthoracic echocardiography (TTE) in POC settings. Transesophageal echocardiography (TEE) allows for interpretable images in nearly all situations regardless of these variables, and the utility of TEE in patients who are critically ill or in cardiac arrest is well documented.^9^, ^10^, ^11^, ^12^, ^13^, ^14^, ^15^, ^16^, ^17^, ^18^, ^19^, ^20^, ^21^, ^22^, ^23^, ^24^, ^25^, ^26^, ^27^, ^28^, ^29^, ^30^, ^31^, ^32^, ^33^, ^34^, ^35^, ^36^, ^37^, ^38^, ^39^, ^40^, ^41^, ^42^ Although some EPs have begun utilizing TEE, it is generally considered an advanced skill outside the reach of the majority of EPs.

Significant literature has described the feasibility and utility of TEE by EPs with advanced ultrasound (US) training.^9^, ^13^, ^17^, ^18^, ^19^, ^36^, ^37^ This led to an American College of Emergency Physicians (ACEP) policy statement on the use of TEE in cardiac arrest.^43^, ^44^ The policy aimed to establish a standard to assist EPs who wish to use TEE in their clinical practice and suggested supervised performance of 10 TEE examinations on live patients or simulated models. Prior to the ACEP policy statement, in 2013, our department began a limited pilot program of focused TEE (F‐TEE) use by our US faculty (those with advanced US training beyond residency). They reported vastly improved imaging in difficult populations, especially with respect to time to image acquisition, image quality, and hands‐free continuous imaging. Given the potential to improve image quality for providers of all skill levels, the group believed that F‐TEE would be even more helpful to the rest of our faculty (those without advanced US training), because they have less training in obtaining high‐quality TTE images in difficult cases. Therefore, we initiated a mandatory training and credentialing program for all of our emergency department (ED) faculty, including full‐time and part‐time faculty as well as all fellows. Our goal was to ensure 24/7 coverage by F‐TEE credentialed faculty to ensure access to F‐TEE for all critical resuscitations.

In this report, we describe our experience with our first 557 cases of F‐TEE after training and credentialing all of our ED faculty. Training consisted of a 4‐h workshop using a high‐fidelity simulator, with no practice on human subjects. To our knowledge this is the first report of mandatory F‐TEE training and credentialing of an entire EM practice group and the first series in which the majority of examinations were performed by physicians without advanced US training beyond EM residency. The primary goals of this retrospective study were to report: (1) feasibility, as determined by the acquisition of interpretable images; (2) quality of those images, compared to traditional POC TTE, particularly by non‐US faculty; and (3) safety.

## MATERIALS AND METHODS

### Study design

This is a retrospective analysis of a continuous quality improvement database of patients who presented to Hennepin County Medical Center between April 26, 2017, and October 1, 2020, who had a TEE performed as part of their ED care. We did not choose a sample size a priori; rather, we chose to include all available cases. There was no algorithm or protocol for TEE use; rather our physicians were free to use it whenever they thought it would be helpful for intubated patients with shock, cardiac arrest, or periarrest. This study was paid for by departmental funds.

### Training and credentialing

All of our full‐ and part‐time ED faculty (*n* = 52) were trained and credentialed in F‐TEE. All were previously trained in POCUS per standard EM guidelines.^5^ The majority of our faculty have no advanced US training beyond residency, and in this study we refer to them as “non‐US faculty.” Ten of our faculty either have extensive experience teaching US (RFR, DP, SJ) or have completed US fellowship training, and they are referred to as “US faculty” in this study.

Prior to the introduction of F‐TEE, we developed a focused curriculum (Table 1) and instructional material modeled after a previously published F‐TEE curriculum.^45^ The majority of ED faculty and fellows (*n* = 40) successfully completed a 4‐h group training course in April 2017 and were subsequently F‐TEE credentialed at our institution. F‐TEE privileging criteria were approved by our hospital's credentials committee, which reviewed our curriculum and our historic experience prior to this department‐wide initiative.

**TABLE 1:** XXX

Topic	Minutes
Critical echocardiographic questions in resuscitation	30
TEE applications; assessment of Global function Mechanical compression effectivity Reversible causes of arrest Procedural guidance	30
Probe mechanics and delivery techniques	20
Limited‐TEE standard views	20
Safe practice	20
Simulation (CAE Vimidex TEE simulator) Image acquisition Image interpretation Tips and tricks	90
Evaluation Probe delivery image acquisition image interpretation Probe removal Safety consideration	30

Abbreviations: TEE, transesophageal echocardiography.

Our curriculum included 2 h of didactic training concentrating on anatomy, probe operation, and placement and details of how to obtain standard views, indications, and contraindications as well as patient safety. We taught just four TEE views; midesophageal four‐chamber, midesophageal long‐axis, transgastric short‐axis, and bicaval. Following didactics and hands‐on instruction and practice, each trainee was subjected to a brief standardized assessment, which consisted of independently placing the transducer and obtaining the four standard views without assistance. All hands‐on training and assessment was performed on a high‐fidelity TEE simulator (CAE Vimedix). No animal or human practice models were utilized.

Primary instructors for our initial group training in April 2017 included three of our department US faculty (RFR, JN, AL), who each had significant previous TEE experience. Also, at the request of our hospital's credentials committee we engaged an outside expert, who published an F‐TEE curriculum, to attend and audit our initial training session.^45^ Subsequent training sessions, for faculty hired after April 2017 (*n* = 12) and for those needing repeat training for recredentialing, were taught by one of our department's three F‐TEE experts.

In addition to the one‐time initial credentialing process, our EM faculty must be recredentialed for F‐TEE every 2 years. Recredentialing requires proof of performance of at least two adequate F‐TEE examinations during clinical practice or repeating the initial 4‐h course curriculum.

### Quality assurance

As part of the credentialing process we instituted a quality assurance (QA) system in which we review all ED F‐TEE studies. All F‐TEE–recorded examinations were internally reviewed by a team of five intradepartmental US experts. Our department has had a robust US QA program for POCUS for more than 20 years. This includes a weekly meeting of the five US faculty who review training images as well as deficiencies, mistakes, and interesting cases. Nearly all F‐TEEs were recorded in real time and stored in our Q‐Path image archiving system for review. In addition, a review of the medical record was performed to assess for procedural complications on an ongoing basis. Safety variables included displacement of the endotracheal (ET) tube, blood on a TEE probe, pneumomediastinum, and esophageal perforation. Major complications were defined as those resulting in significant patient morbidity or mortality. Any such events are reported to our medical staff quality committee as part of the credentialing and oversight process.

### Study variables

A QA database was developed to track all pertinent information regarding all F‐TEE studies. Recorded details including the operator, US training, clinical indications and scenario, and minor and major complications. Demographics were limited to the patient's age, sex, race, height, weight, and body mass index. Information regarding cardiac arrest was limited to out of hospital or in hospital as well as the primary rhythm.

Indications for the examination were separated into three categories: ongoing cardiac arrest, pre‐ or postarrest (periarrest), or shock. Among patients with cardiac arrest, recorded resuscitation variables included whether or not the patient had return of spontaneous circulation (ROSC), whether the arrest occurred before or after the TEE probe was placed, if the probe was in place during CPR, if the probe was in place before a patient rearrested or required defibrillation, and if it was in place during defibrillation. Disposition was recorded as died in the ED, died in the hospital, or survived to hospital discharge.

The physicians performing TEE examinations were divided into two groups: (1) US faculty, those who have completed an US fellowship or have had other advanced US training (RFR, SJ, DP), and (2) non‐US faculty, those with no US training beyond their EM residency.

To ensure validity of potentially subjective data points (timing of TEE placement, resuscitation, and safety variables), three independent abstractors (ESC, CT, TD) were trained, and 10% of all charts in the QI database were randomly selected using the sample_n() function in RStudio (R Core Team, 2021; R Foundation for Statistical Computing) to calculate the interrater reliability (Fleiss' kappa).

Finally, all recorded US images were reviewed for image quality by US faculty (RFR, WS, DL) who graded each as “good” (standard views with important anatomy clearly identified), “fair” (not ideal but sufficient to answer clinical questions), or “poor” (uninterpretable). If a TTE was performed on the same patient, recorded image quality superiority was compared. These reviews were necessarily unblinded as the standard views differ by technique.

### Outcomes

The primary outcome of this study was whether the provider was able to obtain interpretable F‐TEE images during the resuscitation. This was defined as the provider being able to insert the probe, obtain images, and interpret them in the electronic medical record (EMR). Images were not necessarily required to have been saved to be included in the primary outcome and only needed to be interpreted in the EMR for the purpose of this outcome. Secondary outcomes included the quality of images by themselves and compared to TTE as well as the presence of any immediate procedural complications including but not limited to displacement of an ET tube, blood on a probe requiring an intervention (transfusion, endoscopy), and esophageal perforation.

### Data analysis

Demographics and clinical characteristics including faculty training and number of USs performed, indication for examination, and image quality are reported using simple descriptive statistics, with 95% confidence intervals (CIs) around point estimates, when appropriate. Agreement between multiple abstractors was compared using a Fleiss’ kappa. Comparisons between independent proportions are calculated with a z‐statistic, and between groups with chi‐square testing, with two‐sided *p* < 0.05 considered significant. We did not adjust for multiple comparisons.

## RESULTS

**FIGURE 1:**
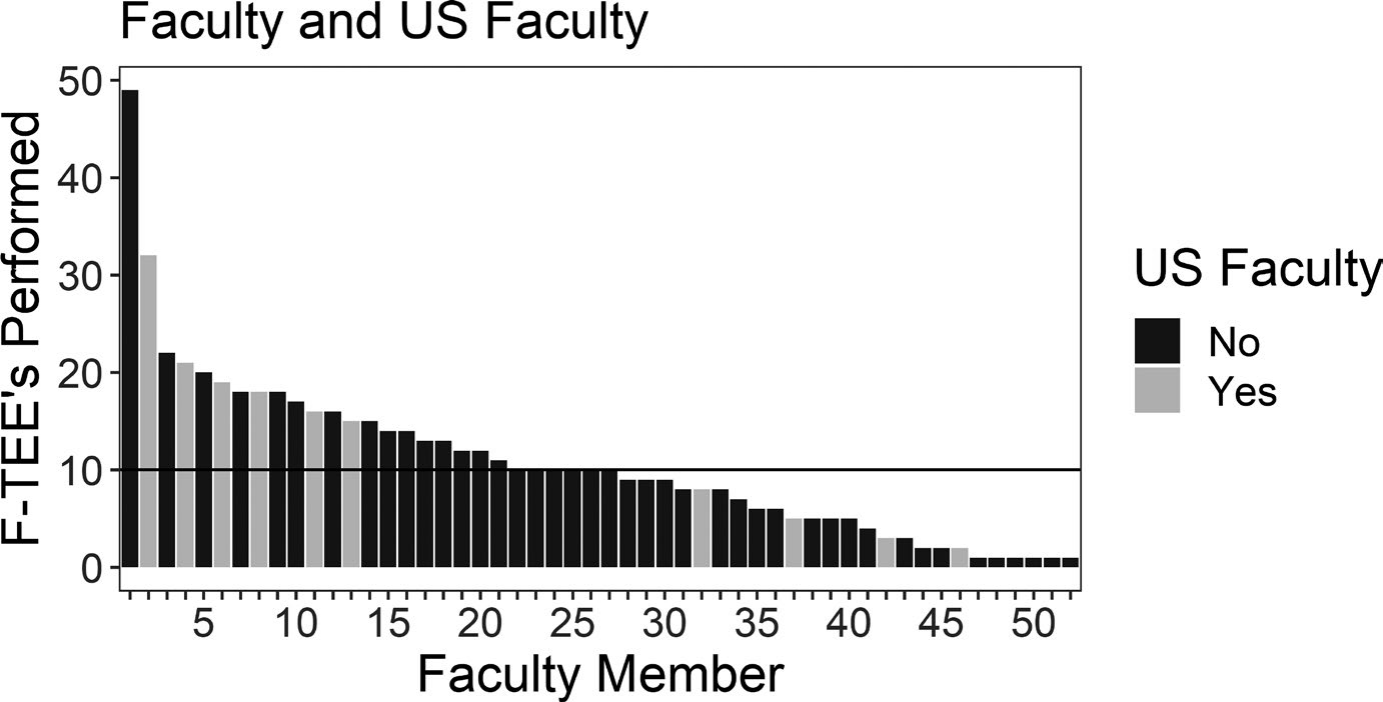
Focused‐TEE examinations performed per faculty member. Non‐US faculty are denoted by black bars and ultrasound faculty are denoted by gray bars. TEE, transesophageal echocardiography; US, ultrasound

Emergency physicians (*N* = 52) attempted a total of 557 F‐TEEs during the 42‐month study period. All F‐TEE images were obtained using a Mindray TE7 system with a P7‐3ts probe. All faculty performed at least one F‐TEE, with a median of 10 (interquartile range [IQR] = 5–15) performed per faculty member. The majority (75%) of examinations were performed by non‐US faculty (Figure 1). Among patients undergoing probe placement, the median age was 58 (IQR = 45–68) and 68.2% were male (Table 2).

**TABLE 2:** Demographics of F‐TEE patients (*N* = 557)

Characteristic	Patients
Age (y), median (IQR)	58 (45–68)
Sex	
Male	380 (68)
Female	177 (32)
Race	
White	280 (50)
Pacific Islander	2 (0.4)
Hispanic	21 (3.8)
Black	163 (29)
American Indian	23 (4.1)
Patient declined	65 (12)
Asian	3 (0.5)
Weight (kg), median (IQR)	86 (72–105)
Unknown	44 (7.9)

Note

Data are reported as median (IQR) or *n* (%).

Abbreviations: F‐TEE, focused transesophageal echocardiography; IQR, interquartile range.

Physicians were able to obtain a viewable image 99% of the time (95% CI = 97.7%–99.6%), which did not differ between non‐US faculty and US faculty (*p* > 0.9). The quality of recorded images was determined to be good in the majority of cases (85.9%, 95% CI = 82.5–88.7), while 9.7 (95% CI = 7.3%–12.6%) were considered fair (suboptimal but interpretable) with only 4.5% (95% CI = 2.8–6.6) considered poor images. US faculty demonstrated a higher proportion of recorded good images (92%) compared to non‐US faculty (84%) and lower proportions of fair and poor images (Table 3; *p* = 0.036).

**TABLE 3:** F‐TEE uses and outcomes (*N* = 557)

Characteristic	Patients
Indication (*n* = 557)	
Cardiac arrest	179 (32)
Periarrest	310 (56)
Shock	68 (12)
Location of cardiac arrest (*n* = 489)	
ED	66 (13)
OHCA	423 (87)
Rhythm (*n* = 489)	
PEA	227 (46)
Ventricular fibrillation	114 (23)
Asystole	111 (23)
Unknown	22 (4)
Ventricular tachycardia	15 (3)
Disposition: cardiac arrest and periarrest (*n* = 489)	
Died in ED	229 (47)
Died In hospital	175 (36)
Survived to hospital discharge	85 (17)
Disposition: shock (*n* = 68)	
Died in hospital	17 (25)
Survived to hospital discharge	51 (75)

Note

Data are reported as *n* (%).

Abbreviations: F‐TEE, focused transesophageal echocardiography; OHCA, out‐of‐hospital cardiac arrest; PEA, pulseless electrical activity.

Transesophageal echocardiography was significantly more likely to yield superior‐quality images compared to TTE in cases when both were recorded (378/408, 92.6%, 95% CI = 90%–95%). US faculty were more likely than non‐US faculty to obtain superior F‐TEE images compared to TTE images (*p* = 0.021).

The most common indication for TEE placement was periarrest (*n* = 310, 55.7%), followed by cardiac arrest (*n* = 179, 32.1%) and shock (*n* = 67, 12.2%; Table 4). Among those who suffered cardiac arrest (the overwhelming indication for TEE placement in this cohort), the majority occurred out of hospital (86.5%), and the majority of primary rhythms were nonshockable (69.1%; Table 4). Consistent with studies of cardiac arrest, a high percentage of patients died in the ED (46.8%). More than half survived to admission, however, with 35.8% of the total cohort dying in the hospital and 17.4% surviving to hospital discharge (Table 4).

**TABLE 4:** F‐TEE outcomes (*N* = 557)

Characteristic	Faculty (*n* = 417)	US faculty (*n* = 138)	*p*‐value^a^
Viewable images	414 (99)	137 (99)	>0.9
TEE image quality			0.036
Good	324/386 (84)	119/130 (92)	
Fair	40/386 (10)	10/130 (8)	
Poor	22/386 (6)	1/130 (1)	
Images superior to TTE	273/301 (91)	105/107 (98)	0.021

Note

Data are reported as *n* (%).

Abbreviations: F‐TEE, focused transesophageal echocardiography; TTE, transthoracic echocardiography; US, ultrasound.

^a^ Pearson's chi‐square test.

Interestingly, in 102 cases the probe was in place during electrical cardioversion/defibrillation (20.9%; Table 5), and there were no reported cases of transducer damage due to electrical shock. In terms of timing of probe placement, F‐TEE was utilized before (32.3%), during (74%), and after (35.2%) ROSC. Even among those with initial ROSC, many patients rearrested or required repeat cardioversion or defibrillation while the probe was in place (19.8%). Finally, the majority of patients (75%) who had TEE placement for the indication of shock survived to hospital discharge.

**TABLE 5:** F‐TEE use during cardiac arrest (*n* = 489)

Characteristic	Patients
ROSC before TEE	
Yes	172 (35)
No	298 (61)
Unknown	19 (4)
TEE during CPR	
Yes	362 (74)
No	114 (23)
Unknown	13 (3)
ROSC after TEE	
Yes	158 (32)
No	312 (64)
Unknown	19 (4)
TEE in place before rearrest/shockable rhythm	
Yes	97 (20)
No	373 (76)
Unknown	19 (4)
TEE probe in place during shock	
Yes	102 (21)
No	362 (74)
Unknown	25 (5)

Note

Data are reported as *n* (%).

Abbreviations: F‐TEE, focused transesophageal echocardiography; ROSC, return of spontaneous circulation.

In terms of safety, we identified only three potential safety issues, and one of these was a preexisting problem that was identified during TEE placement. In one case a patient's ET tube was dislodged during TEE placement. This was immediately recognized and the tube was replaced before oxygen desaturation occurred. A second patient arrived to the ED intubated but with the ET tube cuff inflated above the vocal cords. This was incidentally discovered and corrected when videolaryngoscopy was performed for TEE probe placement. In a third patient, pneumomediastinum was noted after an unsuccessful attempt at TEE probe placement. However, after an extensive review of this case the pneumomediastinum was ultimately attributed to preexisting injuries caused by blunt trauma rather than injury from attempted TEE probe placement (see Discussion). There were no other reported cases of potential esophageal injury and no other cases of significant blood on the TEE probe in this study group. Fleiss' kappa ranged from 0.6 to 1 for data from all tables above.

## DISCUSSION

We are pleased with the results of our first 557 F‐TEE cases and with our faculty's ability to perform and interpret F‐TEE examinations after completing just a 4‐h training program. Over the course of these 42 months, 52 faculty performed F‐TEEs. Previously published reports of F‐TEE have been limited to a small number of highly trained operators who were US fellowship trained and/or completed a significant amount of US training beyond EM residency.^9^, ^13^, ^17^, ^18^, ^19^, ^36^, ^37^ Therefore, the most dramatic finding in our study is that 42 non‐US faculty performed 74.8% of the F‐TEE examinations. We are encouraged that physicians with training limited to their EM residency were able to obtain interpretable images with a success rate of 99.3%, after just 4 h of F‐TEE instruction and no practice on human subjects. The majority of these images were either good or fair (83.9% and 10.4%, respectively). This implies that the “average” practicing EP can reasonably obtain F‐TEE training and begin using this technology in their clinical practice. When these data were presented at a national conference there was concern that these results may not be generalizable because the non‐US faculty in our department might have more US expertise compared to faculty at other residency programs or community EPs. We believe that our results are generalizable, since several of our faculty who trained elsewhere, who work in our department only part time, or who have recently joined our faculty after many years in community practice were all able to perform F‐TEE without any extra training.

Transthoracic echocardiography has been routinely incorporated into management of patients in shock and cardiac arrest with the primary goals of guiding management and identifying treatable causes of arrest.^7^ TEE offers hands‐free continuous cardiac monitoring with exceptional image quality. This enables additional resuscitative advantages not possible with TTE—namely, evaluation for chest compression adequacy, immediate visual feedback of cardiac response to interventions, and identification of impending cardiovascular collapse. F‐TEE is especially useful in determining return of organized cardiac activity, which is immediate, visual, and unequivocal. We find this superior to the indirect and highly inaccurate practice of pulse checks as a marker of ROSC.^46^, ^47^, ^48^ In our study, the most commonly reported indication for performing F‐TEE was continuous cardiac monitoring in the cardiac arrest or periarrest state. F‐TEE was initiated in a significant number of non–cardiac arrest patients (*n* = 68, 12.2%) due to concern for impending cardiovascular collapse.

Anecdotally, our physicians found that patients in the “periarrest” state were particularly well suited for continuous TEE monitoring. They were able to identify sudden decreases in cardiac function prior to loss of pulses or significant variation in blood pressures. This created opportunities to potentially prevent or delay cardiac arrest by administration of bolus vasopressors as well as initiation of CPR at the earliest possible moment. The percentage of patients who may benefit from this type of hyperacute vigilance is unknown and worthy of further study but not captured in the current report.

One of the novelties of this report is that we believe that our department is the first ED in the world to implement an institutionally approved departmentwide credentialing program required of all faculty for resuscitative TEE. The decision to make F‐TEE credentialing mandatory was based on our experience using TEE in the prior 4 years (2013–2017), during which time TEE was performed by our US faculty. It became apparent that limited TEE application was extremely useful and easy to perform and interpret, and required no special skill that could not be easily acquired with readily available simulation technology. Our 4‐h training program focused on a practical approach centered around patient safety and efficient use of faculty time, based on our prior experience as well as reports from other successful training programs.^45^, ^49^ This program included both didactic and hands‐on instruction (Table 1). At the end of the session, all faculty underwent a standardized assessment, to assure proficiency in independently placing the transducer and obtaining the standard resuscitative F‐TEE views. This approach is consistent with the recent trend toward the use of high‐fidelity simulation and a standardized assessment of competency for credentialing rather than documenting an arbitrary number of US examinations.^50^, ^51^, ^52^, ^53^

At our institution, maintenance of F‐TEE privileges requires either performance of two resuscitative F‐TEEs every year or a simulation refresher course with the EM US director. Our EM US faculty perform QA consisting of evaluation of image quality, safety, and operator feedback on all resuscitative F‐TEEs performed in our department. We presented this program to our institutional credentialing committee requesting that our faculty be credentialed to perform resuscitative F‐TEE after completion of this curriculum. The credentialing committee unanimously approved this request. Our department leadership then mandated resuscitative F‐TEE credentialing for all EM physicians. Seventy‐five percent of all resuscitative F‐TEEs were performed by non–US‐trained faculty, with an average of 10 examinations per faculty over 42 months, a testimony to the broad acceptance of this application by our faculty group. Our department had broad support from the hospital administration and from other departments as well as internal funding to purchase a TEE simulator. This level of commitment may not be possible at other institutions, which would limit the generalizability of our success. The revenue generated from billing for F‐TEE and other POCUS examinations eventually enabled our department to purchase 10 TEE transducers, which should encourage other institutions that this practice is financially feasible.

Previously described TEE training programs and guidelines require a number of proctored examinations despite little evidence to support this practice. Most importantly, there are no safety data to support the practice of proctored examinations on real patients. Our program is generally consistent with ACEP guidelines on TEE in cardiac arrest.^43^, ^44^ Those guidelines recommend 10 proctored examinations including probe insertion; however, the guidelines do not specify model type: live, cadaveric, or simulator. With the exception of the initial four providers who piloted our TEE program, no other physicians had any training on a live patient prior to performing their first F‐TEE in the ED. Although this is different from any previously published reports of TEE training, we do not believe that it negatively impacted our ability to use F‐TEE safely and effectively, and it has important implications for the future of F‐TEE in general EM practice.

One of the concerns surrounding use of TEE in the ED is patient safety. When we began using TEE we were most worried about esophageal perforation; however, the three potential safety concerns that we recognized were all airway related. Dislodgement of the ET tube occurred in one case and was quickly recognized and corrected. This may have been a serious complication if it had not been immediately recognized. Airway complications that have been previously reported during TEE include airway obstruction by compression of the trachea or mainstem bronchus, right mainstem advancement of the ET tube, and inadvertent tracheal extubation.^54^, ^55^, ^56^ All of these airway complications are more common in children undergoing TEE, including a 0.5% reported risk of inadvertent tracheal extubation.^55^ Although airway complications are less common in adults, it is important for EPs performing F‐TEE to understand the potential risks, have airway equipment immediately available, and be ready to respond swiftly. Also, EPs should vigilantly monitor all patients undergoing F‐TEE using continuous pulse oximetry, waveform capnography, and any other means available.

The second patient in our study with a potential safety issue could have been easily missed or miscategorized. He arrived with paramedics intubated but with the ET tube cuff inflated above the vocal cords. This was not apparent because there was no significant leak and the capnography waveform was normal. Although it is not our common practice to use videolaryngoscopy to place the TEE probe, we routinely use it for ET intubation and encourage its use for difficult TEE probe placement. In this case it was fortuitous that the provider decided to use videolaryngoscopy on the first try at TEE probe placement, which immediately revealed the misplaced ET tube. Had they tried blind placement of the TEE probe first, the misplaced ET tube would have been attributed to a complication of TEE probe insertion. The lesson here is that EPs should carefully monitor the position and depth of the ET tube before, during, and after F‐TEE examinations and should have a low threshold for using laryngoscopy to ultimately verify correct ET tube placement.

The third patient in our study with a potential TEE safety issue had a pneumomediastinum that was recognized after an unsuccessful attempt at TEE probe placement, but ultimately it was not thought to be caused by the TEE probe. This was a 37‐year‐old woman involved in a motor vehicle collision who arrived via helicopter and lost pulses just prior to arrival. She had pulseless electrical activity (PEA) but her heart could not be visualized with surface US due to extensive subcutaneous emphysema from neck to pelvis. She had a King LTSD airway in place but did not seem to be ventilating adequately, with a poor capnography waveform and end‐tidal CO_2_ (ETCO_2_) less than 15 mm Hg. Bilateral chest tubes were immediately placed and the King airway was removed, but ventilation with a facemask and a laryngeal mask airway was also inadequate. She was then intubated but the capnography waveform and low ETCO_2_ was unchanged. At this point she was still in PEA so providers tried to place the TEE probe to obtain some information about cardiac function, but they could not get the tip of the probe beyond the level of the glottis. They then performed a clamshell thoracotomy, which revealed minimal cardiac movement, no pericardial effusion, and pneumomediastinum; and also that neither lung was inflating with positive pressure ventilation. They then performed a front‐of‐neck open surgical airway and discovered a complete tracheal transection, with the trachea retracted into the upper chest. The trachea was pulled up into the neck and intubated, which resulted in lung inflation but persistently low ETCO_2_ (<10 mm Hg). At that point the patient had been in cardiac arrest for more than 30 min without adequate ventilation and had no cardiac activity so resuscitative efforts were terminated. After a comprehensive review of this case we do not believe that the TEE probe caused any of the patient's injuries or contributed to her poor outcome. This was a difficult case but a good example of how we, appropriately, rely on ultrasound in cardiac arrest and periarrest management, but sometimes need to move on without it when imaging is difficult or impossible. Also, although TEE is useful in critically ill trauma patients, and approximately 10% of patients in this study were trauma patients, there should be some assessment for tracheal and esophageal injury prior to probe placement.

As noted earlier, one of our primary concerns with using TEE in our ED was the risk esophageal injury. The risk of major esophageal bleeding associated with TEE has been documented to be less than 0.1%.^56^ Although we did not have any cases of bleeding in this study, it is a potential concern because the coagulation status of ED patients is usually not known at the time of the F‐TEE examination. However, a recently published series of critically ill patients with coagulopathy or thrombocytopenia who underwent F‐TEE showed that these patients did not have a higher risk of bleeding after the procedure.^57^ A review of the literature for esophageal perforation associated with TEE examinations found four studies including 20,000 patients, with four reported cases of esophageal perforation for a rate of 0.02%.^58^, ^59^, ^60^, ^61^ Importantly, patients in those studies underwent comprehensive TEE examinations, which requires passage of the transducer through and beyond the gastroesophageal junction, with significant manipulation of the transducer tip. Our patients had F‐TEE examinations, with most views obtained from the midesophagus and with minimal mechanical manipulation of the transducer tip, which should make their risk of esophageal perforation significantly lower. We found no cases of suspected or confirmed esophageal perforation in this study.

Another concern that has been raised as TEE becomes more common during cardiac arrest management is whether the TEE probe can remain in place during transthoracic defibrillation. In 2008 Blaivas^13^ published a case series in which he reported four patients who were defibrillated with the TEE probe in place, with no reported harm to the transducers or patients. Since that time there have been no significant data published to support or refute the safety of this practice.^31^, ^62^, ^63^ In verbal communications with colleagues who also use F‐TEE we found that about half remove the transducer during defibrillation and half leave it in place, with no reported negative effects to patients, operators, or equipment. In 2013 when our department US experts started using TEE we decided that we would keep the transducers in place during defibrillation. We made this decision based on anecdotal evidence from colleagues and with the knowledge that TEE transducers have an outer cover specifically designed as insulation from electrical shock. Also, between each use TEE probes undergo an electrical leak test to assure that the insulating cover is intact. Prior to teaching all of our faculty to use TEE in 2017 we defibrillated several patients with the TEE probe in place and with the operator holding the handle, with no reported problems. In this study we had 102 patients who were defibrillated with the TEE probe in place without a single problem reported. Based on our experience we believe that transthoracic defibrillation with the TEE probe in place is safe provided that it has passed the electrical leak test.

Based on our QA process, we found that all providers achieved proficiency inserting the transducer with the appropriate orientation and depth after one to two insertions, which is not surprising given the fact that EPs routinely perform other procedures that involve intubation of the esophagus and trachea. We believe that since EPs have extensive experience with ET and orogastric intubation, the delivery of a TEE probe into the esophagus is well within the performance capability of all EPs without significant additional training.

## LIMITATIONS

The primary limitation of this study is its retrospective nature and the limitations associated with that study design. The primary outcome, the ability to obtain an interpretable TEE image, was unlikely to be heavily influenced by bias. The primary outcome of obtaining a viewable image did not differ significantly between US and non‐US faculty. In secondary, exploratory subgroup analyses, we did not adjust for multiple comparisons because this was not the primary goal of this study. Therefore, it remains uncertain whether a true difference between these groups exists in relationship to image quality, and this remains an area for future investigation. Regardless, the overall success rate of interpretable images remained high in both groups, suggesting that this is a generalizable diagnostic tool for EPs familiar with cardiac US following a brief training course.

As noted, secondary outcomes were more likely to be influenced by bias, due to the subjective nature of perceived image quality and superiority of TEE images over TTE images. However, image quality was assessed by quorum during the QA process, and given the well‐known limitations of TTE images in common scenarios in cardiac arrest (subcutaneous air, limited access to the chest), it is not surprising that TEE images would usually be superior. In terms of other outcomes, while abstractors were not necessarily blinded to the outcomes of the study, inter‐rater reliability was excellent (range = 0.6–1) and was calculated for all variables except demographics, image quality, and TEE image superiority, strengthening the study findings.

One limitation of this study is that only image clips saved by providers were reviewed and captured in the database. In reality, continuous image assessment was often performed throughout resuscitations, for 30–60 min or more, yet only a handful of 6‐s clips were typically recorded, stored, and analyzed. It is likely that in some cases providers obtained clinically useful images in real time but failed to record interpretable clips.

A significant limitation of reporting F‐TEE safety is that it was limited to those who survived long enough to manifest symptoms or signs of a complication, had imaging that might suggest a complication, or who had a postmortem examination. Regardless, our QA team did everything possible to discover complications, including monitoring the hospital event reporting system and attending medical staff quality committee proceedings.

## CONCLUSIONS

This is the largest reported series to date of emergency physician–performed resuscitative focused transesophageal echocardiography. To our knowledge this is the first report of focused transesophageal echocardiography training and credentialing of an entire emergency medicine group. A simple focused transesophageal echocardiography training program using just a high‐fidelity simulator, without practice on human subjects, appears to be a practical and safe method to learn focused transesophageal echocardiography. Emergency physicians without advanced ultrasound training beyond residency performed the majority (75%) of focused transesophageal echocardiography examinations in this series after just 4 h of focused transesophageal echocardiography instruction.

## CONFLICT OF INTEREST

Robert Reardon has worked as a consultant for Mindray Ultrasound USA. The other authors have no potential conflicts to disclose.

## AUTHOR CONTRIBUTIONS

Robert F. Reardon, Dave Plummer, Andrew Laudenbach, Joseph Novik, Thomas Pahl, and Johanna Moore conceived the study and designed the trial. Robert F. Reardon, Elliott Chinn, and Michael A. Puskarich supervised the conduct of the trial and data collection. Robert F. Reardon, Andrew Laudenbach, Andie Rowland Fisher, Will Smoot, Daniel Lee, Joseph Novik, Barrett Wagner, Chris Kaczmarczyk, Emily Thompson, and Teresa Dunphy maintained the database and actively participated in weekly QA. Elliott Chinn and Michael A. Puskarich trained and supervised abstractors. Elliott Chinn, Michael A. Puskarich, Johanna Moore, Craig Tschautscher, and Johanna Moore provided statistical advice on study design and analyzed the data. Robert F. Reardon and Dave Plummer drafted the manuscript, and all authors contributed substantially to its revision. Robert F. Reardon takes responsibility for the paper as a whole.
